# The Development of the Antibacterial Microcapsules of Citrus Essential Oil for the Cosmetotextile Application: A Review

**DOI:** 10.3390/molecules27228090

**Published:** 2022-11-21

**Authors:** Euis Julaeha, Mohamad Nurzaman, Tatang Wahyudi, Sarifah Nurjanah, Nandang Permadi, Jamaludin Al Anshori

**Affiliations:** 1 Department of Chemistry, Faculty of Mathematics and Natural Sciences, Universitas Padjadjaran, Jatinangor 45363, Indonesia; 2 Department of Biology, Faculty of Mathematics and Natural Sciences, Universitas Padjadjaran, Jatinangor 45363, Indonesia; 3 National Research and Innovation Agency, Bandung 40272, Indonesia; 4 Department of Agriculture Engineering, Faculty of Agricultural Industrial Technology, Universitas Padjadjaran, Jatinangor 45363, Indonesia; 5 Study Program of Biotechnology, Postgraduate School, Universitas Padjadjaran, Bandung 40132, Indonesia

**Keywords:** microcapsules, essential oils, antibacterial, Citrus genus, immobilization

## Abstract

Essential oils (EOs) obtained from the Citrus genus were reported to exhibit good antimicrobial activity. Therefore, they can potentially be applied in daily necessities such as textile sectors as antibacterial functional fabric products. However, a packaging technique to retain such volatile and labile active substances is compulsory. In particular, microencapsulation was found to be a common coating technique employed to protect EOs from the effects of light, heat, humidity, stability, and controlled release of active substances. Various microencapsulation techniques have been introduced, but the most widely used method is complex coacervation, as it is simple, inexpensive, and capable of snaring high essential oils. Hence, this review focused on the microencapsulation of the most consumable citrus EOs with complex coacervation methods and their immobilization on commonly carried-out fabrics. In addition, it also discusses the isolation methods of the EOs, their chemical composition, and the mechanism of antibacterial action.

## 1. Introduction

Citrus is a genus of the Family *Rutaceae* [1], consisting of 16 species with many varieties [2,3]. The citrus, known as one of the world’s main fruits produced in several countries with tropical or subtropical climates, is a polyembryonic species cultivated throughout the world, mostly in subtropical or hot tropical areas [4]. Countries such as Brazil, the United States, Japan, China, Mexico, Pakistan, and the Mediterranean are the primary producers of citrus fruits [5]. On the other hand, as a native plant of tropical Asia, citrus is also widely used to treat various diseases [6,7,8]. Therefore, it has important implications in the world of trade and health sciences [9,10].

Various parts of the Citrus plant, such as leaves, fruit, seeds, flowers, rhizomes, bark, and even all plant parts, produce Essential Oils (EOs). However, most of them are obtained from the peel of the fruit with a pungent taste and smell good according to their original plant [11,12]. The EOs are obtained either using various conventional techniques such as cold pressing, solvent extraction, hydrodistillation, or non-conventional methods such as Supercritical Fluid Extraction (SFE), Microwave-Assisted Hydrodistillation (MAHD), and Ultrasound-assisted extraction (UAE) with their advantages and disadvantages. EOs are a complex natural mixture containing about 20–60 chemical components at very different concentrations. Two or three main components characterize EOs at relatively high concentrations (20–70%) compared to others. The main group consists of terpenes, terpenoids, and other aromatic and aliphatic constituents, characterized by a low molecular weight [11]. The main component of this Citrus genus is δ-limonene, followed by β-pinene, α-terpineol, and other components that vary qualitatively and quantitatively. Generally, these significant components determine the biological properties of the EOs.

Citrus oil has an extensive industrial profile in beverages, household products, cosmetics, fragrances, pharmaceuticals, and others [4,9,13,14]. In addition, citrus EOs have attracted much interest from scientists because they have various bioactivities, i.e., antimycotic, antiviral, antioxygenic, antiparasitic, antimicrobial, anti-inflammatory, antiseptic, antidepressant, tonic, carminative, antispasmodic, diuretic, and insecticide [9,15,16,17,18].

The utilization of bioactivity is constrained by the instability and volatile nature of EOs. Therefore, suitable packaging technologies such as microencapsulation are required to cover their weaknesses by protecting the active ingredient with a coating. Several microencapsulation methods have been introduced, including physical methods such as spray drying, lyophilization, supercritical fluid precipitation, and solvent evaporation; physicochemical methods include coacervation and ionic gelation; and chemical methods include interfacial polymerization and molecular inclusion complexation [19,20,21,22]. In the textile field, EOs encapsulation is used for functional fabric applications, i.e., antibacterial, fragrance, mosquito repellent, aromatherapy [23,24,25], anti-UV [26], phase-change material, flame-retardant, and cosmetotextile [27,28].

The development of encapsulation technology increased tremendously in the last decade to stabilize an applicable biomolecule, in particular for antimicrobial functional fabric. The necessity is relevant to the willingness of human beings to have a healthy and comfortable living environment [23,29,30]. The availability of antimicrobial functional fabric used for daily life, such as for cloths, bed sheets, underwear, or for medical purposes, such as a mask or a wound plaster, are expected [31]. Thereby, the discovery of a highly antimicrobial agent that remains stable in an applied environment is required [32]. Out of 16 species of citrus, there were only four (*Citrus aurantifolia* (*C. aurantifolia*), *Citrus nobilis* (*C. nobilis*), *Citrus sinensis* (*C. sinensis*), *and Citrus limon* (*C. limon*)) that have been exploited extensively for their EOs for an antibacterial cosmetotextile application in the form of microcapsules. Therefore, the review focus was based on those four citrus species.

## 2. Potential of Citrus EOs as Antimicrobial Agents

Citrus fruit is in great demand because of its distinctive, refreshing, and high nutritional value [33]. In addition, its peel is a source of EOs, which are formed from one of the metabolic processes resulting in plants. The reaction of various chemical compounds and water has physiological effects, one of which is antibacterial activity [17,34].

Antibacterial activity is interesting because bacterial infections in humans involving mucosal surfaces and skin are a big problem, specifically in developing countries with tropical and subtropical climates. Microorganisms often found on the skin include *Staphylococcus aureus* (*S*. *aureus*), *Escherichia coli* (*E*. *coli*), *Klebsiella pneumonia* (*K*. *pneumonia*), and *Staphylococcus epidermidis* (*S*. *epidermidis*) [35]. Citrus oil’s antimicrobial activity was exploited against medically important pathogens that occur in various infections [7]. The characteristics obtained are related to the function in plants of these compounds [16], which include terpenoids, alcohols, esters, aldehydes, ethers, and ketones [15].

Jafari et al. [36] observed the antimicrobial properties of *C. aurantifolia* EOs against food-borne bacteria isolated from cream-filled cakes and pastries. The cake was contaminated with bacteria that were dominated by *S. aureus* and *Bacillus subtilis* (*B. subtilis*). The EOs of *C. aurantifolia* were able to reduce the growth of these bacteria and the risk of poisoning for those that consume contaminated food. Costa et al. [9] reported the chemical composition of the *C. aurantifolia* EOs and their antimicrobial activity against Gram-positive bacteria *B. subtilis*, *Enterococcus durans* (*E*. *durans*), *Enterococcus hirae* (*E*. *hirae*), *Listeria monocytogenes* (*L*. *monocytogenes*), *S. aureus*, and *S. epidermidis*. Gram-negative bacteria include *Enterobacter cloacae* (*E*. *cloacae*), *E. coli*, *Pseudomonas aeruginosa* (*P*. *aeruginosa*), *Proteus mirabilis* (*P*. *mirabilis*), *Serratia marcescens* (*S*. *marcescens*), and *Salmonella typhi* (*S*. *typhi*) [9]. The results showed that EOs possess good antimicrobial activity, specifically against *S. aureus*, *B. subtilis*, and *S. epidermidis* bacteria. The other study reported by Al-Aamri et al. [15] showed that the antimicrobial activity of EOs of *C. aurantifolia* leaves was higher against *S. aureus* than against *E. coli*. Torimiro et al. [8] found that the EOs of *C. aurantifolia* obtained from fruit peel showed excellent antibacterial activity with a broad spectrum against multidrug-resistant bacteria. Similarly, previous studies proved the antibacterial properties of encapsulated EOs of *C. aurantifolia* and limonene, and EOs against Gram-positive bacteria such as *S. aureus* and *S. epidermidis* and Gram-negative, which include *E. coli* and *K. pneumonia* [37]. Furthermore, a synergistic action was discovered, as evidenced by higher EOs activity than their pure major compound of limonene, and antibacterial activity could be maintained in the microcapsules [38,39,40,41].

Obidi et al. [7] observed the antimicrobial activity of *C. sinensis* EOs against Gram-positive bacteria *S. aureus*, *E. feacalis*, Gram-negative bacteria *P. aeruginosa*, *E. coli*, and *the fungus Candida albicans* (*C. albicans*). It was reported that the *C. sinensis* EOs exhibited good antimicrobial properties and was applicable in treating a disease caused by microorganisms. Atolani et al. [42] also reported that EOs seeds of *C. sinensis* had potential antimicrobial activity for cosmeceutical production. Sharma et al. [43] stated that EOs of *C. sinensis* inhibited the growth of *Aspergillus niger* (*A. niger*), while Li et al. [38] stated that Gram-positive organisms appeared more susceptible to EOs of *C. sinensis* than Gram-negative organisms.

**Figure 1:**
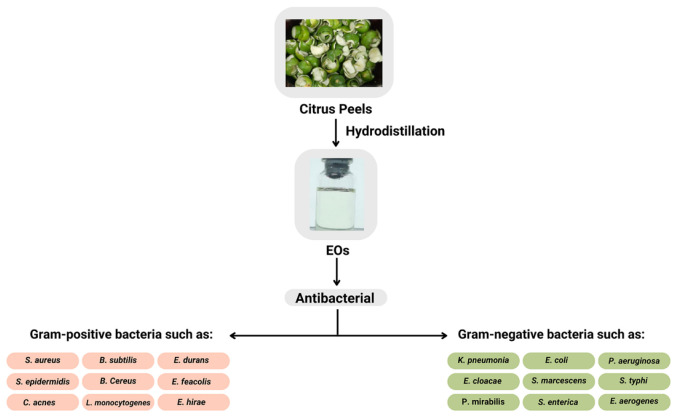
Antibacterial activity of EOs [8,9,15,17,35,36,37].

Himed et al. [44] investigated the antibacterial properties of EOs against nine bacteria, two of which were Gram-positive *Bacillus cereus* (*B. cereus*) and *S. aureus*, and seven of which were Gram-negative: *E. coli P. aeruginosa*, *Salmonella enterica* (*S*. *enterica*), *K. pneumoniae*, *Enterobacter aerogenes* (*E*. *aerogenes*), *Serratia marescens* (*S*. *marescens*), and *Proteus mirabilis* (*P*. *mirabilis*). The EOs of *C. limon* showed an antimicrobial effect against all tested microorganisms. Furthermore, Hou et al. [45] stated that EOs from *Citrus reticulate* (*C. reticulate*) peel were a perfect antibacterial against *Cutibacterium acnes* (*C*. *acnes*) and common microorganisms such as *S. aureus*, *B. subtilis*, and *E. coli*. Antimicrobial activity against the same bacteria was reported by Değirmenci & Erkurt [46] as one of the EOs from the *C. aurantium* flower. Dănilă et al. [47] reported that a mixture of EOs containing terpene alcohol (such as linalool) and δ-limonene could be an effective alternative to antibiotics to treat an *S. epidermidis* infection. Other constituents, such as terpinene-4-ol, α-terpineol, linalyl acetate, neryl acetate, geranyl acetate, and α-pinene were amplified in the presence of limonene (or vice versa). Inhibition of *S. aureus* bacteria by EOs from *C. lemon* leaf was also reported by Fancello et al. [48]. The reported EOs activities are shown in Figure 1.

The numerous reports on EOs activity from the Citrus genus showed that they could potentially be used as antimicrobial agents in the cosmetic textile field. Furthermore, the development of textiles with microcapsules aimed to provide new properties of fabrics with added value. Cosmetotextile is a skincare system that combines cosmetics and textiles through microencapsulation. This textile has cosmetic ingredients inserted into the fabric fibers [23,49], such as aromatic/fragrance textile, cosmetic/dermal functional textile, insect/mosquito-repellent textile, and medical/antimicrobial/antibacterial textiles [23]. Shi & Xin [49] stated that cosmetic textiles act as moisturizing agents, whiteners, fragrances, antioxidants, antimicrobials, energizers and refreshers, and absorbents of sunlight. When the users perform daily activities, the dense microcapsules in the textile material will be released slowly and provide benefits to the body and skin.

## 3. Isolation of the EOs

**Figure 2:**
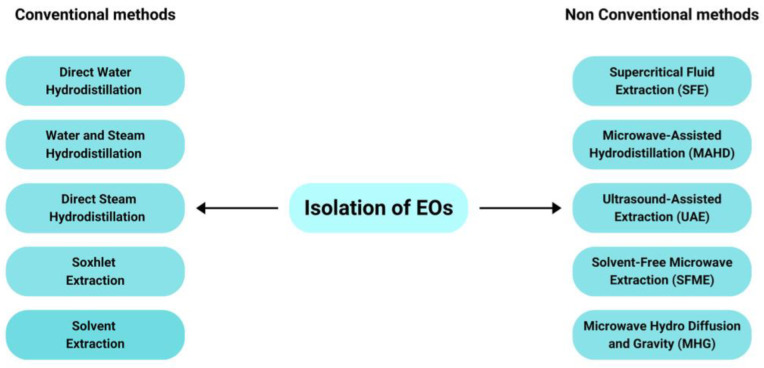
Methods of EOs preparation [50,51].

EOs isolation was conducted using the following conventional and non-conventional methods. Conventional methods include Cold Pressing, Solvent Extraction, Soxhlet Extraction, and Hydrodistillation, while nonconventional methods include Supercritical Fluid Extraction (SFE), Microwave-Assisted Hydrodistillation (MAHD), Solvent-Free Microwave Extraction (SFME), Microwave Hydrodiffusion and Gravity (MHG) [50,51]. For EOs preparation, the most widely used method is hydrodistillation. Based on Table 1, 13 of 15 Citrus samples use the hydrodistillation method for EOs isolation, while the others use MAHD and SFME. The EOs isolation methods are shown in Figure 2.

## 4. Chemical Composition of the Citrus EOs

EOs of the Citrus genus consist primarily of mono- and sesquiterpene hydrocarbons and their oxygenated derivatives, such as alcohols, aldehydes, esters, ethers, and oxides, as well as linear hydrocarbons, alcohols, aldehydes, esters, acids, phenolic compounds, and their derivatives [11]. Terpenoid groups such as monoterpenes, sesquiterpenes, and oxygenated terpene derivatives are EO’s largest group of chemical compounds [52]. The amount of these oxygenated compounds affect the quality of EOs. However, the amount of oxygenated compounds in oil varies and depends on several factors, such as geographic location, climate, species, maturity level, and extraction method [53]. The most important factors are plant genetics and several environmental stress factors (light, moisture content, attack by predators, and pests) [52]. Terpene hydrocarbons do not contribute much to EOs smells because this group of compounds is unstable under heat and light. On the other hand, oxygenated terpenes, mainly consisting of alcohols, aldehydes, and ketones, give a strong taste as a characteristic of EOs [54].

These volatile compounds have different ecological functions, including acting as internal messengers, protecting from herbivorous disturbances, and pollinating insect attractors [55]. In addition, the composition of volatile compounds is the basis for the citrus aroma [56]. The composition of compounds contained in Citrus peel EOs are δ-limonene, β-pinene, α-terpineol, terpinene-4-ol, citronellal, o-cymene, geraniol, β-mirsen, geranyl acetate, β-phellandrene, and citral [4,57,58,59]. The chemical compositions of the five Citrus species are shown in Table 1.

Table 1 shows a summary of the chemical compositions of four Citrus species, such as *C. aurantifolia*, *C. nobilis*, *C. sinensis*, and *C. limon*, with each of the three different recollection places. The plant parts taken were the fruit peel. The methods used most are the hydrodistillation [44,60,61,62,63,64], microwave-assisted hydrodistillation (MAHD) [65], solvent-free microwave extraction (SFME), and microwave-assisted hydrodistillation (MAHD) [66]. GC-MS analysis discovered 101 types of chemical components with varied compositions. Differences in chemical components occur qualitatively and quantitatively, giving each Citrus species a distinctive aroma and strength of activity. Various factors can affect these concentrations, such as harvest time, geographic origin, and regional agro-climatic conditions [38]. Almost all EOs have δ-limonene as the main component with a percentage range of 20–98%. The highest δ-limonene is found in the EOs of *C. sinensis*. Other components that are common in these EOs of Citrus are β-pinene of 0.03–28.4%, β-myrcene of 0.9–4.0%, γ-terpinene of 0.8–16.8%, α-terpineol of 0.1–8.3%, α-Pinene of 0.4–3.1%, terpinene-4-ol of 0.5–4.3%, and Citronellal of 0.1–2.2%. The other components are in minor amounts and only present in a few Citrus species. The chemical composition data in Table 1 prove that the same Citrus species produce different chemical compositions when the recollection place is different.

**Table 1:** Terpenoid compounds make up EOs of several Citrus species.

No.	Chemical Components	*C. aurantifolia*			*C. nobilis*			*C. sinensis*			*C. limon*		
		[58]	[15]	[67]	[58]	[60]	[61]	[58]	[62]	[65]	[58]	[44]	[63]
1.	δ-Limonene	38.9	42.4	39.3	50.1	76.8	81.8	21.7	90.9	98.4	41.4	61.3	75.0
2.	*trans*-Limonene oxide	-	-		-	-	0.3	-	0.01	-	-	0.2	-
3.	β-Myrcene	0.9	1.9	-	1.0	2.4	4.0	-	1.9	1.1	2.4	1.4	-
4.	β-Pinene	26.7	12.6	28.4	3.7	0.8	-	15.4	-	0.03	14.2	9.7	-
5.	α-Pinene	-	3.1	1.5	-	1.1	2.1	0.8	-	0.4	-	1.5	-
6.	α-Terpineol	8.3	1.6	2.4	4.1	-	0.2	5.4	0.1	-	1.7	0.4	-
7.	1-Terpinenol	-	0.04	-	-	-	-	-	-	-	-	-	-
8.	4-Terpineol	-	0.4	-	-	-	-	-	-	-	-	-	-
9.	Terpinene-4-ol	4.3	-	2.0	1.5	0.7	0.5	1.8	-	-	-	-	-
10.	Terpinolene	-	-	-	-	0.7	0.4	-	0.1	-	-	0.2	-
11.	α-Terpinene	-	0.37	-	-	-	-	-	-	-	-	-	-
12.	γ-Terpinene	-	15.4	0.8	-	8.2	6.1	-	1.2	-	16.8	3.8	-
13.	Geranial	-	-	2.1	-	-	-	-	0.1	-	-	-	-
14.	Geranyl acetate	2.6	0.6	0.6	-	-	0.2	1.2	-	-	1.7	-	0.3
15.	Geraniol	1.3	0.6	7.5	0.8	-	-	-	-	-	1.3	-	-
16.	Citronellal	-	-	-	2.2	-	-	1.8	0.1	-	-	0.3	-
17.	β -Citronellal	-	0.1	-	-	-	-	-	-	-	-	-	-
18.	Citronellol	-	-	-	1.9	-	-	-	-	-	-	0.6	-
20.	Citral	3.6	-	-	0.6	-	-	-	-	-	2.7	4.2	8.1
21.	Z-Citral	-	2.0	-	-	-	-	-	-	-	-	-	-
22.	E-Citral	-	1.8	-	-	-	-	-	-	-	-	-	4.4
23.	p-Cymene	-	-	-	-	-	-	1.4	-	-	-	0.1	-
24.	β- Ocymene	-	0.3	-	-	-	-	-	-	-	-	0.1	-
25.	o-Ocymene	1.9	1.3	-	-	-	-	-	0.3	-	2.3	-	-
26.	α-Phellandrene	0.9	0.1	-	-	-	-	-	-	-	1.4	-	-
27.	Neral	-	-	5.3	-	-	-	-	0.1	-	-	-	-
28.	Nerol	0.9	-	-	-	-	-	-	-	-	-	-	-
29.	Z-Nerodilol	-	-	0.6	-	-	-	-	-	-	-	-	-
30.	Neryl acetate	-	2.2	-	-	-	-	-	0.02	-	-	-	1.4
31.	β-Bisabolen	1.0	-	-	-	-	-	-	-	-	1.7	-	0.6
38.	Linalool	-	0.6	-	-	0.3	0.9	-	0.9	-	-	0.4	-
40.	Linalool oxide	-	-	-	-	-	-	-	-	-	-	-	0.4
41.	Trans-linalool oxide	-	-	-	-	-	-	-	-	-	-	-	0.4
43.	3-Carene	-	0.5	0.5	-	-	-	-	0.1	-	-	-	-
44.	Carvone	-	-	-	-	-	0.3	-	-	-	-	-	-
45.	α-Farnesene	-	-	-	-	0.51	0.3	-	-	-	-	-	-
46.	(Z)-β-Farnesene	-	0.1	0.4	-	-	-	-	-	-	-	-	-
47.	(E)-β-Farnesene	-	-	1.5	-	-	-	-	-	-	-	-	-
48.	α-Thujene	-	1.0	-	-	0.2	0.4	-	-	-	-	0.2	-
49.	Sabinene	-	2.1	-	-	-	1.2	0.5	-	0.07	-	-	-
50.	δ-elemene	-	0.2	-	-	-	-	-	-	-	-	-	-
51.	β-Elemene	-	0.3	-	-	-	-	-	-	-	-	-	-
52.	γ-Elemene	-	0.1	-	-	0.4	0.3	1.2	-	-	-	-	2.2
53.	Humulene	-	0.1	0.1	-	-	-	1.2	-	-	-	-	-
54.	trans-Carveol	-	-	-	0.7	-	-	-	-	-	-	-	-
63.	Germacrene-B	-	0.1	-	-	-	-	-	-	-	-	-	-
64.	Germacrene D	-	0.2	-	-	-	0.1	-	0.1	-	-	-	0.2
67.	Methyl chavicol	-	-	-	-	3.7	-	-	-	-	-	-	-
68.	δ-Cadinene	-	-	-	-	-	0.3	-	-	-	-	-	-
70.	Camphor	-	0.01	-	-	-	-	-	-	-	-	-	0.26
71.	Trans-carveol	-	-	-	-	-	-	-	-	-	-	-	0.20
72.	Camphene	-	0.14	-	-	-	-	-	-	-	-	-	-
73.	Cis-Carveol	-	-	-	-	-	-	-	-	-	-	-	0.20
77.	α-caryophyllene	-	-	-	-	-	-	-	-	-	-	-	-
78.	Trans-caryophyllene	-	0.9	-	-	-	-	-	-	-	-	-	-
79	Trans-α-bergamotene	-	1.4	0.4	-	-	-	-	-	-	-	-	0.4
81.	Myristicin	-	-	-	-	-	-	-	-	-	-	-	0.8
Isolation Method		HD	HD	HD	HD	HD	HD	HD	-	MAHD	HD	HD	HD
Recollection place (Country)		IDN	TWN	MYS	IDN	TUR	IRN	IDN	CHN	VNM	IDN	DZA	IDN

Most of the bioactivity from EOs is determined by one or more of its main components. EOs of *C. aurantifolia* exhibit important antimicrobial activity against bacteria, specifically Gram-positive and *Candida* sp. [9]. However, EOs activity sometimes cannot be attributed to one of its main components but is a synergistic effect of several other chemical compounds [67]. For example, Costa et al. [9] stated that limonene has antibacterial activity. However, its antibacterial activity is lower than its antifungal activity. The presence of linalool compounds can increase the antibacterial activity of limonene. The presence of α-pinene, linalool, and β-pinene compounds in *C. aurantifolia* leaf EOs supports the synergistic performance of the limonene [15]. EOs activity of *C. aurantifolia* is higher than limonene against Gram-positive *S. aureus*, *S. epidermidis*, and Gram-negative *K. pneumoniae*, but in contrast, against *E. coli* [68].

## 5. The Mechanism of Antimicrobial Action of the EOs

EOs act selectively on vital microbial functions with minimal or no effect on host function. Different EOs act in different ways. The factors determining EOs activity are composition, functional groups in the active components, and their synergistic interactions [69]. An ideal antibacterial agent should have selective toxicity, meaning that a drug is harmful to the parasite but not harmful to the host.

The antimicrobial action mechanism of EOs is explained using cell wall degradation and disruption of the cytoplasmic membrane or membrane proteins, which causes cytoplasmic leakage, and cell lysis, and ultimately leads to cell death [38,69]. Furthermore, Hu et al. [70] stated that the antibacterial mechanism of EOs occurs by disruption of the membrane with low molecular weight and highly lipophilic components, which pass quickly through cell membranes and cause interference with bacterial cells. EOs can also significantly decrease ergosterol, a significant sterol component, and maintain cell function and integrity [71].

**Figure 3:**
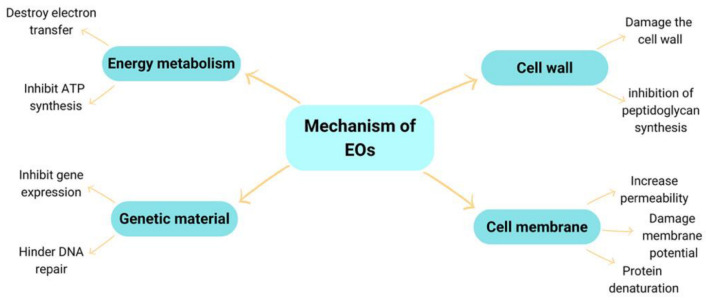
Antimicrobial action mechanism of EOs [69,70,72].

The mechanism of bacterial inhibition by EOs was also presented by Salazar et al. [72] and is suspected of damaging the cell membrane; hence, the cell experiences leakage and changes in the morphology. Furthermore, the provision of these EOs can cause the release of Ca^2+^ and K^+^ ions. The antimicrobial activity of EOs is due to the presence of terpenoids. The aqueous phase is replaced by terpenoids, causing membrane expansion, increased fluidity and permeability, protein disturbance, respiratory inhibition, and altered ion transport processes. Due to the lipophilic nature of EOs, they interact by changing the permeability of cell membranes in microorganisms, causing death [72,73]. The possible antibacterial mechanism of EOs is shown in Figure 3.

## 6. Complex Coacervation Methods

Several methods of bioactive agent encapsulation have been introduced, but choosing the most appropriate method is not easy, because a variety of factors must be considered. Jyothi et al. [74] and Martins et al. [75] also state that the method used for microencapsulation of EOs depends on the source of the plant and the difference in characteristics of each plant’s EOs causes the methods used to be different. Valle et al. [24] propose that the selection of microencapsulation methods must pay attention to some of the following factors: the nature of the core; the smoothing material of possible interactions that occur between the core, surfactant, and shell material; the size of the microcapsule; the mechanism of transfer or release of the core to be achieved; toxicity; and economic factors.

Microencapsulation is a process that involves the formation of a thin layer arranged from polymers [40]. This technology can package material in the form of solids, liquids, or gases into small particles called microcapsules. Encapsulation of EOs in small capsules can prevent oxidation usually triggered by moisture, metal ions, oxygen, and heat [76]. In addition, encapsulation aims to increase the stability and soluble power of the material and regulate the rate of release of active substances, thus contributing to the increase in the shelf life of the product [77,78,79], immobilizing or limiting contact between certain parts of a system and protecting active compounds [79,80], preventing chemical reactions between two active species, and modifying density, color, shape, or photosensitivity [81].

Table 2 is a summary of the results of research conducted by researchers for the microencapsulation of EOs from several species of the genus Citrus using various microencapsulation methods. Some of them have immobilized the fabric for application into a functional fabric. Campelo et al. [82], encapsulated LO using spray-drying methods and evaluated the effect of different dextrose equivalent values on emulsion characteristics. The results showed that maltodextrin with a higher dextrose-equivalent (DE) value had a lower viscosity, resulting in a smaller droplet size. The bioactive compounds contained in LO can maintain high antioxidant activity.

Liu et al. [86] used the orifice method to encapsulate sweet orange EOs using a chitosan-alginate and CaCl_2_ cross-linker. Previously, terpenes in specific limonenes were removed using molecular distillation, since terpenes are easily oxidized to carvol and carveol in the presence of heat and light. The evaluation results showed efficient encapsulation with good morphology and microstructure up to 87.34% yield, when the concentration of CaCl_2_, sodium alginate and ratio of the shell to the core were 2.0%, 2.5%, and 5:1. The release profile of terpeneless sweet orange oil from microcapsules can be well explained by Higuchi’s equations.

Lin et al. [33] conducted a feasibility study on the use of chitosan-based citrus oil microcapsule with different emulsifiers like Tween 20, Tween 40, Tween 60, Tween 20/Span 80 (1:1), Tween 20/SDBS (1:1), and Span 80. This type of emulsifier affects the insertion rate, release rate, and size of microcapsule droplets. Tween 60 shows the best embedding rate and minimum particle size. The rheological properties of the microcapsule and the mechanical and physical properties of stand-alone coatings are also affected by emulsifiers. Emulsifiers with the right HLB tend to have lower nano-level droplet sizes.

Ramos et al. [93] compared two methods of encapsulation in particles containing EOs from orange, namely vacuum spray drying and conventional spray drying, taking into account the physical aspects and storage conditions. The polymers used were maltodextrin 24% (*w*/*w*) and modified starch 8% (*w*/*w*). The results showed that the particle produced by the vacuum spray dryer had lower porosity and lower water adsorption than the spray-dried particles. Particles produced by both processes exhibited amorphous characteristics and no interaction between the wall material and encapsulation oil was observed.

Souza et al. [88] demonstrated encapsulation of limonene by the method of coacervation using fixed concentrations of chitosan and surfactant Lutensol ON 30 (BASF) of 0.50, and three different NaOH concentrations of 0.50, 1.00, 1.45 wt%. Microcapsules with dimensional character, microcapsule shape, and volatility are best generated at NaOH concentration of 1.45 wt%. However, microcapsules produced at a concentration of 1% of NaOH weight were produced in higher amounts and showed very similar results in terms of volatility. The microcapsule is compressed in non-woven fabric cellulose by the padding method, and NaOH concentration stabilization efficiently controls the rate of release of encapsulated active substances, which shows great potential for application in anti-mosquito fabrics.

Li et al. [89] investigated the microcapsule sweet orange essential oil (SOEO) in body weight and colon microbiota in obese mice induced by a high-fat diet. The results showed the SOEO microcapsule loses weight and increases the relative abundance of Bifidobacterium (genus level) in the colon microbiota, protecting the intestinal barrier and lowering colon endotoxin levels by increasing the content of Bifidobacterium.

Rodrigues et al. [83] successfully produced polyurethane-urea microcapsules using the interfacial polymerization method, with limonene as the active substance for textile applications. SEM micrographs showed effective adhesion between microcapsules and textile fibers and also confirmed the morphology and size of caterpillars. Upon dry cleaning test, the impregnated microcapsules lose their core up to 24% in the first cycle and 97% in the fifth cycle. Further abrasion test toward the fibers, reduce their encapsulated limonene up to 40 and 60% at 3000 and 9000 cycles respectively.

Out of the 14 studies in Table 2, the most widely used method is coacervation [32,68,85,86,88,90], ahead of interfacial polymerization [83], orifice [83], emulsion-ionic gelation [91], and spray drying [93]. This is because the method is simple and inexpensive [24], does not require high temperatures [40] and can snare oil up to 99% [76]. However, the deficiency of this preservation is that it only occurs with a certain pH range, colloidal concentration, and limited electrolyte concentration, so the process must be under optimum conditions [30,94,95,96]. Therefore, many researchers study factors that influence the success of microencapsulation with this method. Coacervation is a technique that involves hardening the polymers around the nucleus by changing physicochemical characteristics, such as temperature, ionic strength, pH, and polarity. Complex coacervation occurs through interactions between two opposingly charged polymers, usually proteins and polysaccharides. During the complex coacervation process, the carboxyl groups in polysaccharides and amino groups of proteins will interact to form amide groups [77,97].

**Figure 4:**
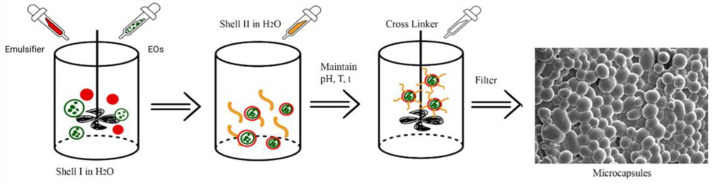
Complex coacervation methods [32,40,41,83,85,98].

There are three basic steps in complex coacervation. The first is the dispersion of the core material (EOs) into an aqueous polymer solution, followed by deposition of the tapping material into the core particles. The second step is the addition of salt or adjusting the variable of pH, temperature, or dilution of the medium. The third step is stabilization of the microcapsule through process of cross-binding, destruction, or thermal treatment [83,98]. This complex coacervation method is influenced by several factors and parameters including pH, temperature, comparison of the shell and capsule core [85], and cross-binding agent [32]. An illustration of a complex coacervation procedure as shown in Figure 4.

## 7. Microcapsule Shell

The type of shell used in microencapsulation processes must have characteristics such as being non-toxic, not reacting with protected substances, having the ability to enclose and hold active materials, providing maximum protection to coating materials [90,99], being chemically compatible, strong (not fragile), flexibility (soft and plastic), impermeability (as a release control under certain conditions), tasteless, non-hygroscopic, low-viscosity, economical, dissolvable in aqueous media or in suitable and stable solvents. In addition, a microcapsule shell must be widely usable in microcapsule manufacturing methods [83,100].

As shown in Table 2, the widely used polysaccharides are alginate and arabic gum, while the proteins are gelatin and chitosan. Alginate, which is obtained commercially from brown seaweed, can cross-link under mild conditions with divalent cations, such as Ca^2+^, inducing a resistant gel formation [24]. This biopolymer is widely used because of its good solubility in water and low viscosity at high concentrations [101], biocompatibility, and non-toxicity, making it widely used for applications in the fields of food, cosmetics, veterinary medicine, pharmacy, and medicine [75].

Gum arabic is a natural resin derived from the exudate of Acacia Senegal and Acacia seyal trees. This polymer consists of polysaccharides and glycoproteins that have an average molecular weight between 300 and 800 kDa. Its composition is 95% polysaccharide on a dry base and 1–2% of different protein species [24]. Arabic gum is a negatively charged polyelectrolyte widely used in industry due to its high solubility and low viscosity at high concentrations and its good emulsification and microencapsulation properties [102]. The polymer is extensively applied in various types of industries, such as food, drinks, confectionery, and in pigments, either as a stabilizer or coagulant, or to avoid such crystallization and agglomeration processes [85,103].

Gelatin is a protein derived from denatured collagen consisting of 18 amino acids, which have functional characteristics in terms of biocompatibility, biodegradability, emulsifier capacity and good hardening ability, water-solubility, high stability, and non-carcinogenicity [9,24]. The disadvantage of gelatin is the need for the use of cross-binding agents such as formaldehyde or glutaraldehyde, where such ingredients are toxic and harmful to humans [101].

Chitosan obtained by the alkaline deacetylation of chitin N is a hydrophilic, biocompatible, and biodegradable polysaccharide, with low toxicity. Chitosan is widely used as an encapsulation agent for several applications, such as food processing, biomedicine and pharmaceutical, wastewater treatment, and textiles, alone or in combination with polysaccharides or other proteins to improve the properties of the shell [24].

Alginate and gelatin are excellent at coacervation methods due to their slight aggregation, small particle size, and easy dispersibility [101]. In textile applications, chitosan alginate microcapsule has been used to encapsulate antimicrobial peptides for applications of cotton coatings [24].

## 8. Cross-Binder

Cross-bonding is a chemical bond formed between polymer chains. Cross-bonding is one way to control the process of releasing active compounds where these cross-bonds serve to harden and stabilize polymers. There are several methods for cross-binding: (i) High-temperature heating, a physical cross-binding method in which the polymer is heated at a temperature of >90 °C and under vacuum. When it is heated, the water is removed and causes a condensation reaction between the carboxyl group and the amine group, resulting in an intermolecular cross-bond. This process has the disadvantage of being able to denaturize polymers made of proteins; (ii) Ultraviolet radiation, which causes the formation of free radicals. When adjacent radicals react, covalent bonds, or cross-bonds, are formed between two polymer molecules; (iii) Crosslink agents: cross-bonding between polymers causing microcapsule structures to become sturdy [75,104].

Cross-bonding consists of chemical modifications aimed at binding polymer chains through reactions between specific reactive sites present in polymer structural units and some cross-binding reagents [24]. A good cross-binding agent should contain a reactive end to the specific functional group (primary amine, sulfhydryl, etc.) in the polymer. Examples of cross-linking agents that are often used include formaldehyde, glutaraldehyde, calcium chloride [105], and tannic acid. Properties such as mechanical resistance, swelling, permeability, chemistry, and thermal stability as well as the rate of release of encapsulated substances are strongly influenced by cross-bonding. Depending on the nature of the cross-binding, the main interactions involved in tissue formation are covalent or ionic [24,106,107].

Glutaraldehyde and formaldehyde are the cross-binding agents most commonly used to form covalent bonds between aldehyde groups and amines. During the cross-binding process, the aldehyde group of glutaraldehyde/formaldehyde forms an amine covalent bond (Schiff base) with the gelatin/chitosan amino group and an acetal bond with the hydroxyl group. Glutaraldehyde and formaldehyde have drawbacks, i.e., toxicity, making it necessary to develop new cross-binders that are more environmentally friendly [24].

Tannic acid (C_76_H_52_O_46_)_,_ is a naturally occurring plant polyphenol. Tannic acid is a gallic ester of D-glucose in which the hydroxyl group of carbohydrates is sterilized with a gallic dimer. Some of its phenolic hydroxyl groups can interact with biological macromolecules through hydrogen bonding and hydrophobic interactions. Therefore, tannic acid has been used as a hardening substance [24].

Dong et al. [108], compared the use of formaldehyde and transglutaminase cross-binders in the manufacture of the microcapsule, with gelatin and arabic gum, and nuclei in the form of peppermint oil. The results showed formaldehyde was a better cross-binding agent than transglutaminase. Alvim & Grosso [106] made microcapsules using the cross-binder of glutaraldehyde and transglutaminase, gelatin, and arabic gum, with oleoresin nuclei. The results showed that glutaraldehyde cross-binders were better than transglutaminase. Noppakundilograt et al. [109] succeeded in making microcapsules with CaCl_2_ as a cross-binder, with an alginate and eucalyptus oil as the core.

## 9. Emulsifier

Emulsifiers are one of the factors that affect the successful formation of microencapsulation. Emulsifiers serve to affect the formation of microcapsules especially the average diameter and stability of the dispersion. HLB (Hydrophilic-*Lipophilic Balance)* is the value for measuring the efficiency of the emulsifier used.

The higher the number of HLB the more hydrophile a surfactant and the lower the number of HLB the lipophile of a surfactant. Emulsifiers with HLB numbers greater than 10 have a higher affinity for water (hydrophilic), while those with HLB numbers greater than 10 have a higher relative affinity for oil (lipophilic) [110]. The most widely used emulsifiers are tween 80 and span 80.

Tween-80 (*Polyoxyethylene sorbitan monooleate*) is a nonionic surfactant, non-toxic, environmentally friendly, biocompatible, and low commercial price [101]. Produces oil and water emulsions with a smooth texture, stable at high electrolyte concentrations and changes in pH. Soluble in water, ethanol, and ethyl acetate, it is insoluble in liquid paraffin and polyhydric alcohols.

Span-80 (*Sorbitan monooleate*) is a nonionic surfactant with characteristics of ivory yellow, a liquid like a viscous oil, and a distinctively sharp smell. Its solubility is insoluble but dispersed in water, mixed with alcohol, insoluble in propylene glycol, soluble in almost all mineral and vegetable oils, and slightly in ether. Span-80 hydrophobic is a surfactant commonly used in the formation of emulsions/microemulsions and shows good results in improving fuel properties and having a low toxicity [24].

## 10. Microcapsules Immobilization onto Textile Materials

Microcapsules can be applied to woven, non-woven, knitting, or garment fabrics. Substrates can be wool, silk, cotton, hemp, or synthetic fibers such as polyamide or polyester, or its mixture [111]. The widely used textile material is cotton fabric as shown in Table 2, this type of fabric comes from natural fibers that are a comfortable place for microorganisms to grow and develop. This is because natural fiber textile materials can retain moisture. Cotton fabric has very comfortable properties because it can absorb sweat, while human sweat itself is a suitable shelter for bacterial growth. The presence of carbohydrates in cotton fiber acts as an intake of nutrients and energy sources for microorganisms. These microorganisms can cause damage to fabrics and are also a source of pathogens. The process of finishing fabrics with antimicrobial microcapsules can better protect the wearer against the spread of microbes, bacteria, and diseases than protect the quality and durability of textile materials.

Several methods of applying microcapsules on textile substrates such as bath exhaustion, padding, dry curing pad, dipping, chemical grafting [24,111], printing, coating, spraying, or immersion [23]. For all these methods, a cross-binder, such as acrylic, polyurethane, or silicone, is required to bind the microcapsules to the fabric and hold it during washing. Microcapsules on fabric using cross-binding substances must be prepared under appropriate reaction conditions and have high heat-resistance performance, slow-release rates, and good morphology [23].

**Figure 5:**
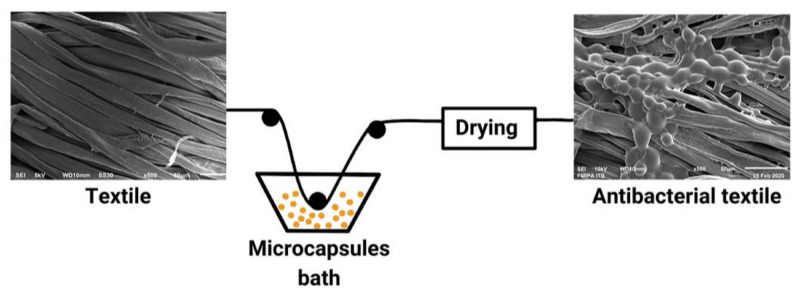
Pad-dry methods [23,24,37,111,112].

According to Valle et al. [24], the method of microcapsule application in fabric depends on the chemical and fabric used, as well as the available machinery. Chemicals that have a strong affinity with the surface of the fiber can use the batch process with exhaustion after the dyeing process of the fabric is complete. As for chemicals that do not have an affinity with fiber, the method uses a variety of continuous processes by passing the fabric into a microcapsule solution and continuing with certain mechanical processes that involve either soaking the textile in a final chemical solution or applying the final solution to the fabric in some mechanical way. The most widely performed microcapsule fixation on fabrics is pad-dry-cure, with the following steps: In the padding process, textile fabrics are continuously passed into microcapsule liquor. After the coating of the fabric by the liquor, the textile fabric is then dried and cured (heated to cause chemical reactions) in each machine separately. The drying process can be done by heat convection, contact with heated metal surfaces, infrared radiation, microwave or high-frequency waves, combustion, and vacuum. The cure or microparticle fixation process is done at a higher temperature than the drying process [37]. An illustration of the immobilization of microcapsules on cloth by the pad-dry method is shown in Figure 5.

Applications for antibacterial functional fabrics of the core material of species *C. aurantifolia* have been made by Sharkawy et al. [32] with limonene nuclei as the main components of *C. aurantifolia* EOs, with synthetic lime oil cores [90], and with EOs of *C. aurantifolia* cores [68]. Sharkawy et al. [32] applied the antibacterial properties of limonene and vanilla to cotton fabric. Limonene and vanillin were encapsulated by a complex coacervation method using chitosan/gum Arabic shells with a cross-binder of tannic acid. Microcapsules were immobilized on the fabric by esterification reactions of citric acid and monobasic sodium phosphate monohydrate (as a catalyst) followed by thermofixation and curing. Antibacterial properties were tested on *S. aureus* and *E. coli.* The results showed free microcapsules and that processed cotton fabrics can maintain antibacterial activity. Wijesirigunawardana & Perera [90] encapsulated synthetic lime oil with chitosan and arabic gum. Microcapsules were immobilized on cotton fabric by a grafting method using succinic acid binder and the antibacterial properties were tested against *E. coli*, *B. cereus*, *S. typhimurium*, and *S. aureus*. It was concluded that the lime oil’s antibacterial activity can be maintained in the microcapsule [90]. Julaeha et al. [37] encapsulated EOs of *C. aurantifolia* using gelatin alginate shells. Microcapsules were immobilized on a cotton fabric by a pad-dry-cure method using citric acid binder followed by an antibacterial activity assay against Gram-positive bacteria *S. aureus* and *S. epidermidis* as well as Gram-negative *E. coli* and *K. pneumoniae.* In addition, microcapsule activity was compared to the EOs of *C. aurantifolia* and limonene. The results showed the synergistic effect of the components contained in *C. aurantifolia* EOs. The activity of *C. aurantifolia* EOs matched the positive control of ampicillin and limonene, while the microcapsule was able to maintain its antibacterial activity [37].

## 11. Future Prospects

Research into the potential of essential oils of the Citrus genus as antibacterial agents has shown good potential for applications in the textile field, for example for medical/antimicrobial/antibacterial textiles [23]. As was mentioned by Gokarneshan et al. [31], the antibacterial properties of natural materials can open up opportunities for antimicrobial coating applications in the medical field. Valle et al. [24] also stated that new interests are needed to develop antibacterial and antiviral properties for technical applications in the fields of medicine, agriculture, geology, architecture, and others. In textile materials, Personal Protective Equipment (APD) is indispensable not only in a hospital environment but also for a public pandemic situation.

**Figure 6:**
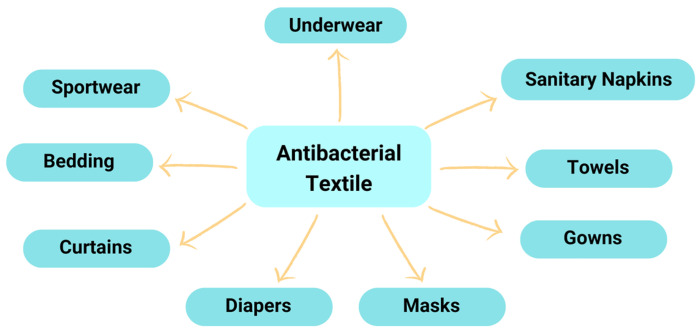
Application of antibacterial textile [23,24,31].

The choice of antimicrobial agents depends on the end-use, requirements, and how the antimicrobials work. There are two ways an antimicrobial agent can work: the first is through chemical binding to the surface of a fabric. Microbes are killed by encapsulated agents when they come into contact with the fabrics. Second, an antimicrobial agent is only physically attached to a fiber and dissolves or diffuses. Application of antibacterial properties of EOs can be used in various products, as shown in Figure 6.

## 12. Conclusions

Essential oils from citrus plants have the potential to be used as antibacterial agents that have a great opportunity to be applied in an encapsulated form overlaid on textile materials. Such products can be produced for medical textiles, such as antibacterial masks, antibacterial plasters, antibacterial sanitary napkins, for Personal Protective Equipment, and for household and functional clothing, including antibacterial bed linen, antibacterial underwear, antibacterial clothing, and others. This challenges researchers to develop and maximize the work of antibacterial microcapsules made from essential oil cores, especially from the genus Citrus; such work includes improving the yield and quality of essential oils, increasing microcapsule yield, controlled release of essential oils, improving microcapsule stability, the effectiveness of microcapsule immobilization in textile materials, etc.

## Figures and Tables

**Table 2:** Microencapsulation of essential oils of the genus Citrus and immobilization on textile materials.

No.	Microencapsulation					Immobilization			Activity	Ref.
	Core Material	Shell Material	Crosslinker	Emulsifier	Method	Fabric	Binder	Method		
1.	Limonene oil	Polyurethane-urea	PEG 400, EDTA Hydrazine	Polyvinylalcohol	Interfacial polymerization	Wool/polyester	Baypret USV	Foulard	-	[83]
2.	Limonene oil	ethyl cellulose	-	-	Simple coacervation	Cotton	1,2,3,4-butanetetracarboxylicacid (BTCA)	grafting	-	[84]
3.	Sweet orange oil	Soybean protein isolate-gum Arabic	-	PEG 2000 PEG 4000 Maltodextrin Sucrose	Complex coacervation	-	-	-	-	[85]
4.	Sweet orange oil	Chitosan- sodium alginate	CaCl_2_	-	Complex coacervation	-	-	-	-	[86]
5.	Lemon fragrance	-	-	-	Commercial microcapsules	60% wool, 38% poly-ester, and 2% elastane.	polyacrylate	Pad-Dry-Cure	-	[87]
6.	Limonene oil	Chitosan	-	Lutensol ON 30 (BASF)	Simple coacervation	Cellulose non-woven	-	Padding	-	[88]
7.	Limonene and vanillin	Chitosan-gum Arabic	Tannic acid	PGPR 4150 Span 85	Complex coacervation	Cotton	Citric acid	Grafting	*S. aureus* *E. coli*	[32]
8.	Lime oil	Whey protein- maltodextrin	-	-	Orifice	-	-	-	Antioxidant	[82]
9.	Citrus oil	Chitosan	Tween 20, 40,60 Tween 20/Span 80 (1:1) Tween 20/SDBS (1:1) Span 80	-	Emulsion-ionic gelation	-	-	-	-	[89]
10.	Lime oil	Chitosan-gum arabic	-	-	Complex coacervation	Cotton	Succinic acid	Dipped	*E. coli*, *B. cereus*, *S. typhimurium*, and *S. aureus*	[90]
11.	Sweet orange oil (*C. sinensis*)	β-cyclodextrin	-	-	Inclusion encapsulation	-	-	-	diet-induced obese	[91]
12.	Lemon oil (*C. limon*)	melamine-formaldehyde	-	-	Complex coacervation	woven silk	acrylic	pad-dry-cure	-	[92]
13.	Lime oil	Gelatin-sodium alginate	Glutaraldehyde	Tween 80	Complex coacervation	Cotton	Citric acid	Pad-Dry-Cure	*S. aureus* *S. epidermidis E. coli* *K. pneumoniae*	[37]
14.	Orange oil	Maltodextrin-modified starch	-	-	Vacuum spray drying Conventional spray drying,	-	-	-	-	[93]

## Data Availability

The study did not report any data.

## Sample Availability

Samples of the compounds are not available from the authors.
